# Rv0180c contributes to *Mycobacterium tuberculosis* cell shape and to infectivity in mice and macrophages

**DOI:** 10.1371/journal.ppat.1010020

**Published:** 2021-11-01

**Authors:** Delphine Payros, Henar Alonso, Wladimir Malaga, Arnaud Volle, Serge Mazères, Sébastien Déjean, Sophie Valière, Flavie Moreau, Stéphanie Balor, Alexandre Stella, Lucie Combes-Soia, Odile Burlet-Schiltz, Olivier Bouchez, Jérôme Nigou, Catherine Astarie-Dequeker, Christophe Guilhot

**Affiliations:** 1 Institut de Pharmacologie et de Biologie Structurale (IPBS), Université de Toulouse, CNRS, UPS, Toulouse, France; 2 Institut de Mathématiques de Toulouse, UMR5219, Université de Toulouse, CNRS, UPS, Toulouse, France; 3 INRAE, GeT-PlaGe, Genotoul, Castanet-Tolosan, France; 4 Plateforme de Microscopie Électronique Intégrative (METi), Centre de Biologie Intégrative (CBI), CNRS, Toulouse, France; National Institutes of Health, UNITED STATES

## Abstract

*Mycobacterium tuberculosis*, the main causative agent of human tuberculosis, is transmitted from person to person *via* small droplets containing very few bacteria. Optimizing the chance to seed in the lungs is therefore a major adaptation to favor survival and dissemination in the human population. Here we used TnSeq to identify genes important for the early events leading to bacterial seeding in the lungs. Beside several genes encoding known virulence factors, we found three new candidates not previously described: *rv0180c*, *rv1779c* and *rv1592c*. We focused on the gene, *rv0180c*, of unknown function. First, we found that deletion of *rv0180c* in *M*. *tuberculosis* substantially reduced the initiation of infection in the lungs of mice. Next, we established that Rv0180c enhances entry into macrophages through the use of complement-receptor 3 (CR3), a major phagocytic receptor for *M*. *tuberculosis*. Silencing CR3 or blocking the CR3 lectin site abolished the difference in entry between the wild-type parental strain and the Δ*rv0180c*::*km* mutant. However, we detected no difference in the production of both CR3-known carbohydrate ligands (glucan, arabinomannan, mannan), CR3-modulating lipids (phthiocerol dimycocerosate), or proteins in the capsule of the Δ*rv0180c*::*km* mutant in comparison to the wild-type or complemented strains. By contrast, we established that Rv0180c contributes to the functionality of the bacterial cell envelope regarding resistance to toxic molecule attack and cell shape. This alteration of bacterial shape could impair the engagement of membrane receptors that *M*. *tuberculosis* uses to invade host cells, and open a new perspective on the modulation of bacterial infectivity.

## Author summary

The epidemic efficiency of tuberculosis bacilli is determined by their capacity to transmit via aerosol. Currently, the bacterial functions that favor *Mycobacterium tuberculosis* seeding in the lung of naïve host remain mostly unknown. Here we implemented a genome-wide approach to identify *M*. *tuberculosis* mutants deficient for seeding and early replication in the lung of mice. In addition to genes known to encode virulence factors, we identified three genes not previously described. We used complementary approaches to characterize the phenotype of a *M*. *tuberculosis* mutant with insertion within the *rv0180c* gene. We found that this mutant is impaired for seeding in the lung of mice and for invasion and replication in human macrophages. In macrophages, the defect relies on a lack of engagement of CR3 receptor. Although we did not detect any difference between the wild type strain and the *rv0180c* mutant with regard to potential CR3-ligand, we found that the bacterial cell envelope is altered in the *rv0180c* mutant. Our study provides new insight into bacterial genes required for early interaction of *M*. *tuberculosis* with the host and perspective to understand the bacterial functions enhancing infectivity.

## Introduction

*Mycobacterium tuberculosis*, the etiologic agent of human tuberculosis (TB), is spread in the population through the air [1]. Infectious droplets are expelled by TB patients during respiratory maneuvers, such as breathing, talking and coughing, and are inhaled by close contacts [2,3]. Small particles that successfully pass the anatomical barriers of lung airways are deposited in alveolar sac where macrophages and epithelial cells engulf TB bacilli starting a novel infectious cycle [4,5]. This aerosol transmission route has largely contributed to make TB the most important infectious disease of our times with an estimated 10 million new TB patients and 1.4 million TB deaths in 2019 [6].

Transmission is intimately associated with i) the severity of disease in the donor host and the pathogen*-*induced tissue lesions associated with bacterial release into lung airways (such as granulomas with central liquefactive necrosis and cavities), ii) the production of infectious droplets by coughing TB patients, and iii) the bacterial capacity to transit through the respiratory system of contacts, to avoid early clearance and to seed in lung alveoli to initiate replication and infection (a last step defined as the infectivity).

There are several studies indicating that the transmission is determined in part by the genetic background of the strain. Indeed, the pioneering work of Riley and colleagues [1] indicated that patients having comparable sputum positivity exhibited wide variability in their capacity to transmit. This initial observation was confirmed by other studies where some phylogenetic lineages or isolates showed lower transmission after controlling for clinical and demographic confounding factors [7–9]. By whole-genome sequencing and evolutionary convergence analysis of a strain collection from the Netherlands, Nebenzahl-Guimaraes *et al*. [8] identified 5 loci (*espE*, *PE-PGRS56*, *Rv0197*, *Rv2813-Rv2814c*, and *Rv2815-2816c*) under positive selection in highly transmissible isolates and found that mutations in these genes modulated the inflammatory response *in vitro*. However, the function of these genes remains to be established. It is also unclear whether they impact the bacterial capacity to generate lesions (such as cavities) associated with bacterial release in lung airways, to be expelled from the lung of TB patient (*via* induction of cough) or to transit in lung airways and to seed in a new host. This limited number of studies indicates that there is a lack of knowledge with regard to the bacterial factors and adaptations favoring the transmission of *M*. *tuberculosis*.

Numerous studies established that the infectious dose is extremely low and one colony forming unit (cfu) is sufficient to initiate an infection in animal models [10–12]. These observations suggest that *M*. *tuberculosis* has evolved functions and adaptations to counter both the physical and immunological barriers from the host lung. Such adaptations and functions are major determinants for the epidemic capacity of *M*. *tuberculosis* and its long-term association with the human host but remain mostly unknown.

Here we implemented a genome-wide TnSeq approach to search for bacterial functions important for seeding and early replication of *M*. *tuberculosis* in the lung of mice. We found several genes already known as major virulence factors and identified new candidates of unknown function, among which the *rv0180c* gene. We used complementary approaches to explore the role of *rv0180c* and its contribution to the bacterial infection in the lungs.

## Results

### Genes required for infectivity in mice

**Fig 1:**
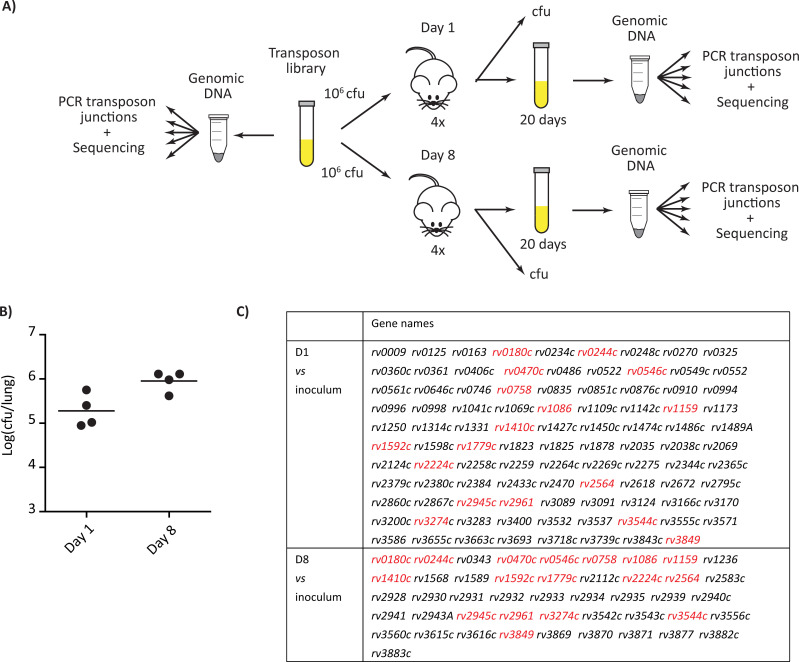
Identification of genes required for *M*. *tuberculosis* seeding in the lung of mice. (A) Scheme of the TnSeq strategy used. BALB/c mice were infected intranasally with 10^6^ cfu of our H37Rv-derived transposon library. At 1 day and 8 days post-infection, four mice were sacrificed. Part of the lung and spleen homogenates were plated to evaluate bacterial load. The remaining part of the organ homogenates was used to inoculate a culture used for DNA extraction, transposon-junctions amplification and TnSeq analysis. (B) Bacterial load in the lung of mice after 1 and 8 days. Each dot corresponds to a different mouse and the mean of the four mice is indicated. (C) Genes in which insertion were underrepresented in mice after 1 or 8 days of infection in comparison to the inoculum. The list reports only the gene detected both using the ESSENTIAL and TRANSIT softwares; in red, the genes common to the two time points.

**Fig 2:**
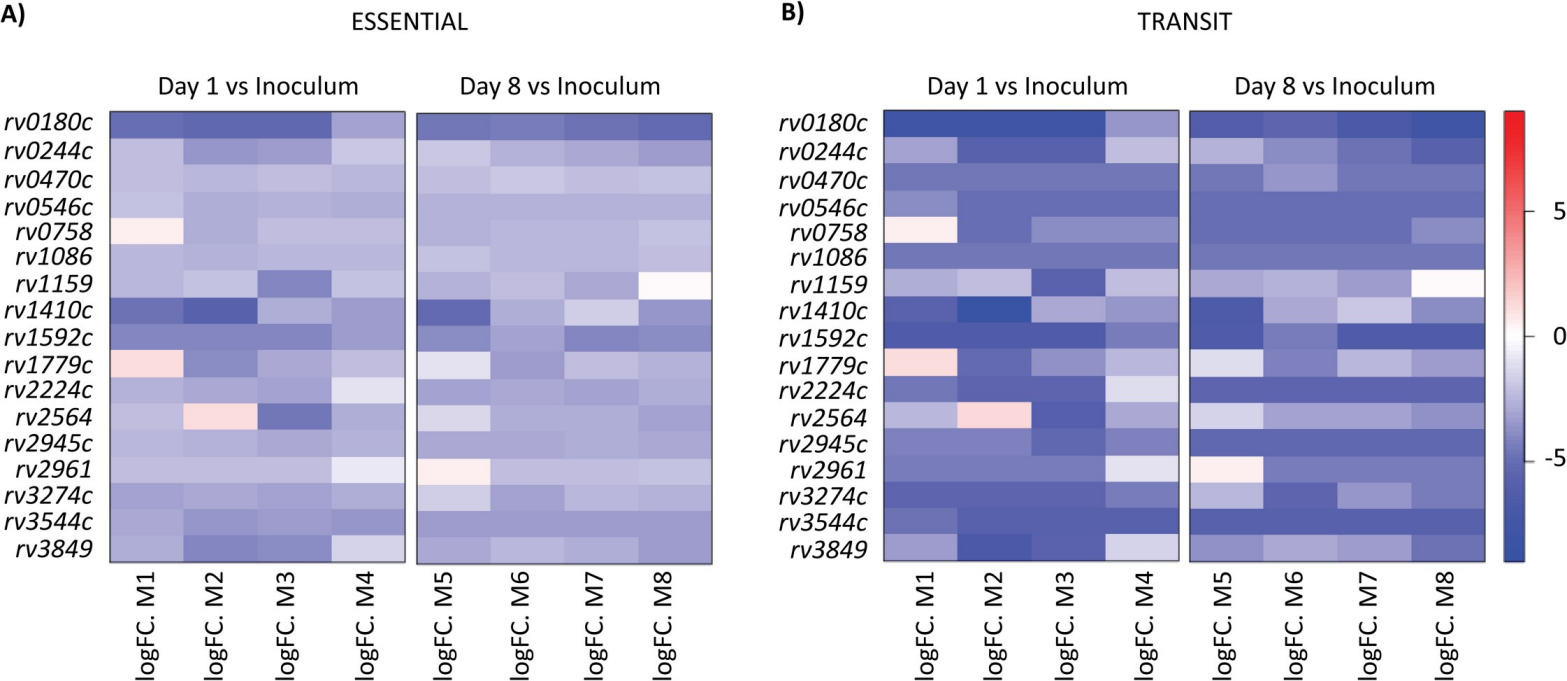
Heat-map for the number of reads in the selected genes after 1 or 8 days of infection for each mouse in comparison to the inoculum. The 17 selected genes were identified using both ESSENTIAL and TRANSIT softwares. The color scale corresponds to the log fold change of the number of reads.

Our goal was to unravel the *M*. *tuberculosis* functions required for the initiation of infection in the lungs. We first generated a transposon mutant library of the *M*. *tuberculosis* H37Rv strain using the phagemid ϕMycomarT7, which contains a mariner-derived transposon on a thermosensitive bacteriophage, as described by Sassetti et al. [13]. This library contains approximately 7x10^4^ mutants. To identify genes important for *M*. *tuberculosis* infectivity, we infected BALB/c mice with the library of H37Rv transposon mutants (Fig 1A). We instilled 10^6^ cfu intranasally to 8 mice and recovered live bacteria from the lungs after 1 day and 8 days (4 mice for each time-point). The bacterial load for each mouse was evaluated by plating serial dilution on solid medium. We obtained around 1.9x10^5^ and 1x10^6^ cfu in the lungs at day 1 and 8 post infection respectively (Fig 1B). These results suggest that the library diversity (less than 7x10^4^ different mutants) was preserved (S1A Fig). The expansion of the bacterial population in the lung during the 8 days period was consistent with previous results using the same inoculation route [14]. To identify the H37Rv mutants exhibiting a deficient capacity to survive the initial encounter with the host, we compared the mutant distribution in the inoculum and in the populations recovered from the lung of mice at days 1 or 8 post-infection using the TnSeq approach [15–17] and the ESSENTIAL and TRANSIT analytical methods [18,19]. The Pearson’s correlations were between 0.9 and 0.99 for technical replicates and between 0.51 to 0.8 at day 1 and between 0.86 and 0.89 at day 8 for biological replicates. We selected only genes identified by both ESSENTIAL and TRANSIT methods and we found 98 genes with significant different number of reads at day 1 and 46 genes with significant different number of reads at day 8 in comparison to the inoculum (Figs 1C and S1B). Candidates identified at day 8 but not day 1 could represent mutants with impaired capacity to grow *in vivo* once implanted in the lungs. The biological meaning of genes identified at day 1 but not day 8 is less obvious, but may represent mutants delayed in their capacity to regrow *in vitro* after the brief environmental stress encountered in the lung. We reasoned that the genes important for bacterial seeding and capacity to initiate the infection should be found in both lists. Therefore, we focused on the 17 common genes (Figs 2 and S1B).

This list contains several genes already identified as important for the virulence of *M*. *tuberculosis*. This includes: three genes *rv0244c* (*fadE5*), *rv3274c* (*fadE25*) and *rv3544c* (*fadE28*) encoding acyl-CoA dehydrogenases required for growth on cholesterol and predicted to be involved in cholesterol side-chain β-oxidation and degradation [20,21]; two genes, *rv3849* (*espR*) and *rv0758* (*phoR*) encoding key regulators for *M*. *tuberculosis* virulence [22–25]; and *rv2224c* (*hip1*) required for acid stress resistance [26]. We also found several genes involved in the biosynthesis of cell envelope components: *rv2945c* (*lppX*) encoding a lipoprotein important for the translocation of the major lipid phthiocerol dimycocerosate [27,28]; *rv0470c* (*pcaA*) required for the formation of cyclopropane ring in mycolic acids [29]; *rv1086* encoding the enzyme required for the formation of decaprenyl diphosphate, a carrier molecule required for the biosynthesis of cell wall and lipoarabinomannan (LAM) [30]; *rv1159* (*pimE*), a gene of the phosphoinositolmannoside (PIM) pathway [31] and *rv1410c* (*p55*) encoding an efflux pump required for the translocation of various lipoglycans (PIM, triacylglycerol and LAM) [32]. Our analyses also picked three genes, *rv0546c*, *rv2564*, *rv2961* encoding conserved proteins with similarities to S-D-lactoylglutathione methylglyoxal lyase, the ATP-subunit of a glutamine ABC transporter (GlnQ), and a transposase, respectively. Finally, we also detected several genes of unknown function, *rv0180c*, *rv1779c* and *rv1592c*, among which we decided to focus on *rv1080c* because mutants with insertion in this gene were the most severely attenuated in our TnSeq analysis (Fig 2). Both *rv0180c*- and *rv1592c*-insertional mutants were also detected as highly attenuated at 10 and 45 days post-infection in another TnSeq study [17]. The *rv0180c* gene is conserved in mycobacterial pathogens, such as *M*. *marinum*, *M*. *ulcerans*, and the highly degenerated *M*. *leprae*, but we did not identify orthologue in fast growing mycobacteria such as *M*. *smegmatis*, *M*. *phlei*, *M*. *abscessus* or *M*. *fortuitum* (S2A Fig). This gene encodes a 452 amino acid protein with no assigned function. It is predicted to contain 6 transmembrane domains and exhibits weak similarities with ABC transporter. Two studies showed that peptides derived from Rv0180c bind human phagocytes and epithelial cells [33] and that antibodies raised against Rv0180c inhibit bacterial entry into monocytes [34]. These studies did not explore the role of Rv0180c in the biology of *M*. *tuberculosis* but suggested that it may contribute to host-pathogen interaction.

### Rv0180c contributes to the infectivity of *M*. *tuberculosis* in mice

To further address the role of *rv0180c*, we inactivated this gene by deletion of an internal fragment and insertion of a kanamycin resistance cassette to generate the Δ*rv0180c*::*km* mutant (S2B and S2C Fig). We also generated a complemented strain carrying an integrative plasmid with *rv0180c* under the control of the promotor pblaF*. Finally, we transferred in these strains a replicative plasmid carrying the *gfp* gene.

Using these tools, we first evaluated the impact of the *rv0180c* inactivation on the bacterial growth rate in standard liquid medium. Growth of the Δ*rv0180c*::*km* mutant, the Δ*rv0180c*::*km* complemented strain and the parental H37Rv wild-type strain (WT) were monitored by measuring the optical density over a period of 17 days. In these conditions, we detected no difference (S2D Fig).

**Fig 3:**
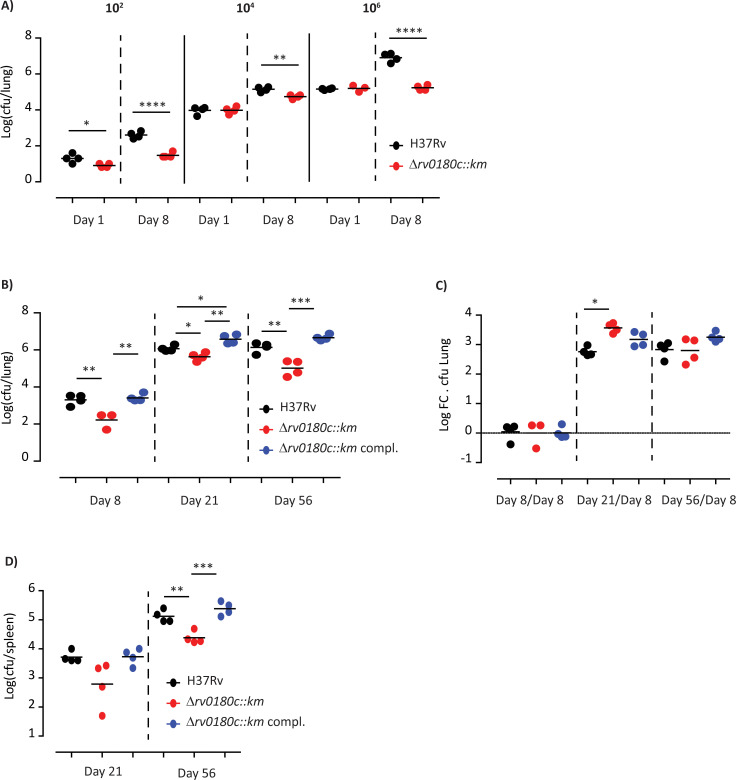
The Δ*rv0180c*::*km* mutant is impaired for implantation in the lung of mice. (A) Parental *M*. *tuberculosis* H37Rv (WT) and the Δ*rv0180c*::*km* mutant were used to infect mice intranasally using different infectious dose (10^2^, 10^4^, and 10^6^ cfu per mouse). At 1 and 8 days after infection, four mice for each group were sacrificed and the bacterial load in the lungs was evaluated by plating. Results are shown as the Log_10_ cfu for individual mice and the mean. These results are representative of two experiments. *p<0.05, **p<0.01, ****p<0.001 by one-way analysis of variance (ANOVA) followed by the Bonferroni post hoc test. (B, C, D) Parental *M*. *tuberculosis* H37Rv (WT), the Δ*rv0180c*::*km* mutant, and the Δ*rv0180c*::*km* complemented strains were used to infect mice intranasally using 10^3^ cfu per mouse. At 8, 21 and 56 days after infection, four mice for each group were sacrificed and the bacterial load in the lungs (B) and spleen (D) was evaluated by plating. Results are shown as the Log_10_ cfu for individual mice and the mean. These results are representative of two experiments. (C) To evaluate the impact of the Δ*rv0180c*::*km* mutation on bacterial multiplication, we calculated the Log Fold Change (Log FC) of bacterial load at days 21 or 56 over day 8. *p<0.05, **p<0.01, ****p<0.001 by one-way analysis of variance (ANOVA) followed by the Bonferroni post hoc test.

Then we investigated the impact of the mutation on the infectivity of *M*. *tuberculosis*. Mice were infected intranasally with various infectious doses (10^2^, 10^4^, or 10^6^ cfu) of the WT strain or the Δ*rv0180c*::*km* mutant and we evaluated the bacterial load in the lungs after 1 or 8 days of infection. For the three doses, we counted a lower number of cfu for the Δ*rv0180c*::*km* mutant than for the WT strain at 8 days post-infection (Fig 3A). These results confirmed the TnSeq analyses and demonstrated that mutation in *rv0180c* reduced the infectivity of *M*. *tuberculosis* during either competition or independent infection experiments. We also verified that genetic complementation restored the bacterial load to WT level (Fig 3B), indicating that deletion of *rv0180c* gene was indeed responsible for the observed phenotype.

We next evaluated whether the *rv0180c* disruption impairs the capacity of *M*. *tuberculosis* to replicate in the lungs. Mice were infected intranasally with 10^3^ cfu of either H37Rv, the Δ*rv0180c*::*km* mutant, or the complemented strain and we monitored the bacterial loads in the lung and spleen at 8, 21 and 56 days post-infection (Fig 3B, 3C and 3D). We confirmed that the number of cfu is decreased by more than 1 log for the Δ*rv0180c*::*km* mutant in comparison to the WT strain or complemented strain at 8 days post-infection. At later time points, we observed that the attenuation was maintained with 0.5 and 1 log less cfu for the Δ*rv0180c*::*km* mutant than for the parental or complemented strains at 21 days and 56 days post-infection respectively. However, the ratio of bacterial load at days 21 or 56 post-infection over the initial load (day 8) revealed that the bacterial expansion was similar or higher for the Δ*rv0180c*::*km* mutant than for the parental or the Δ*rv0180c*::*km* complemented strains (Fig 3C). Therefore, the Δ*rv0180c*::*km* mutant is not impaired for multiplication in the lung of mice. In the spleen, we also found that the Δ*rv0180c*::*km* is attenuated at 21 and 56 days post-infection (Fig 3D) but this difference may be a consequence of the differential bacterial load in the lung.

If a reduced transit to the lung alveoli explains the reduced infectivity observed with the Δ*rv0180c*::*km* mutant then changing the infection route should mask this phenotype. To address that question, we performed one experiment where groups of BALB/c mice were infected intravenously with 10^4^ cfu. Again, we observed lower cfu count for the Δ*rv0180c*::*km* than for the WT in the lung at 8 days post-infection whereas the bacterial loads were similar for the two strains in the spleen (S3 Fig). At day 21 post-infection, we found lower cfu for the mutant strain than for the WT in both organs. This result is consistent with that obtained by Zhang et al. [17] using a TnSeq approach showing that mutants with insertion in *rv0180c* are substantially underrepresented in the lungs of C57BL/6 mice 10 days and 45 days after intravenous infection.

From these results, we concluded that the *rv0180c* gene is required for *M*. *tuberculosis* infectivity in the lungs of mice. The attenuation is primarily seen for the Δ*rv0180c*::*km* mutant during the very early time points as *in vivo* growth is not restricted between day 8 and day 21. In addition, this effect is independent from the inoculation route, indicating that the lung alveoli is not required to observe the phenotype associated with *rv0180c* deletion.

### Rv0180c is required for entry of *M*. *tuberculosis* in human macrophages via complement receptor 3 (CR3)

We then explored the phenotype of Δ*rv0180c*::*km* mutant at the cellular level. We focused on macrophages because they are the first cellular target of *M*. *tuberculosis* during early infection and this encounter between the pathogen and its host cell plays a crucial role in the outcome of the infection [5]. In addition, macrophages are the main cellular niche for intracellular growth and persistence of *M*. *tuberculosis* during all phases of TB [35].

**Fig 4:**
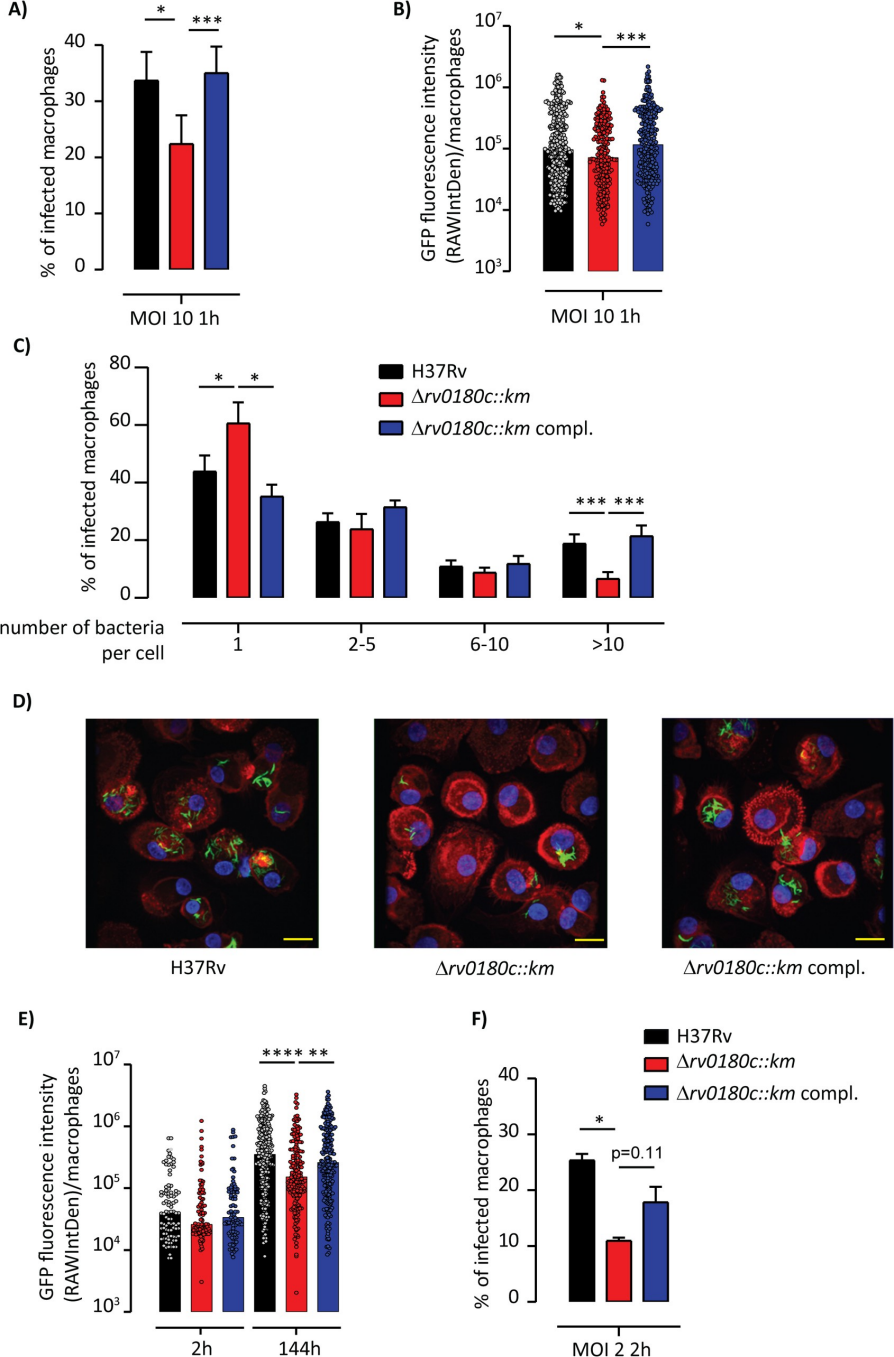
The Δ*rv0180c*::*km* mutant is impaired in its ability to invade hMDMs. (A-C) hMDMs were incubated with GFP-expressing strains (parental H37Rv, the Δ*rv0180c*::*km* mutant, or the Δ*rv0180c*::*km* complemented strain) at MOI 10:1 for 60 min at 37°C and processed for analysis by fluorescence microscopy. (A) Vertical bar plots indicated the percentage of hMDMs having ingested at least one bacterium, (B) Vertical scatter plots represented the distribution of GFP fluorescence intensity (reflecting bacterial loads) in individual hMDMs with 100 to 500 cells counted per condition, (C) Percentage of infected hMDMs as the function of the number of ingested bacteria. Ingested bacterial clumps are counted in the group of more than 10 bacteria (>to 10). The values are medians (B) or means +/- SEM (A, C) calculated from 6 donors. (D-F) hMDMs were incubated for 2h at 37°C with strains at MOI 2:1. After being infected, cells were washed and further incubated with fresh medium containing serum. At 2 h and 144 h post-infection, hMDMs were fixed and processed for confocal fluorescence microscopy. (D) Representative immunofluorescence images showing 144h-post-infected hMDMs. Bacteria are GFP-positive (green) and cells were stained for nuclei with DAPI (blue) and F-actin with rhodamine-phalloidin (red). Scale bar, 10μm. (E) Vertical scatter plots representing distribution of GFP fluorescence intensity in individual infected hMDMs with 100 to 500 cells counted per conditions (F) Vertical bar plots represented the percentage of infected hMDMs after 2h of infection. The values are medians (E) or means +/- SEM (F) calculated from 4 donors. For (A-C, F), the statistical significance was evaluated using the one-way ANOVA followed by the Bonferroni post hoc test. For (B,E), the statistical significance was evaluated using the Kruskal-Wallis rank sum test followed by Dunn’s multiple comparison test; *p<0.05, **p<0.01, ***p<0.001, ****p<0.0001.

We therefore investigated the infectivity of Δ*rv0180c*::*km* in our model of macrophages, the human monocyte-derived macrophages (hMDMs) [36]. We incubated cells with GFP-fluorescent bacteria at multiplicity of infection of 10 bacteria for 1 macrophage (MOI 10:1) for 1h at 37°C. The macrophages were then fixed and prepared to count the number of infected cells and the number of bacteria per macrophage using confocal fluorescence microscopy. We also determined GFP fluorescence intensity per infected cell, which reflects the intracellular bacterial load (Fig 4A, 4B and 4C). We found a 33% reduction in the number of cell harboring at least one fluorescent bacteria for the Δ*rv0180c*::*km* in comparison to the WT or genetically-complemented strains (Fig 4A). We also observed that the distribution of GFP fluorescence intensity is slightly shifted to the lower values for the Δ*rv0180c*::*km* mutant in comparison to the two other strains (Fig 4B). Most macrophages infected by WT, Δ*rv0180c*::*km* mutant or Δ*rv0180c*::*km* complemented strains contained between 1 and 5 bacteria per cell but there are more cells containing more than 10 bacteria for the WT and Δ*rv0180c*::*km* complemented strains than for the Δ*rv0180c*::*km* mutant (Fig 4C). Finally, we found that the mutant infected fewer hMDMs than did the other strains whatever the time of infection (60 to 180min) (S4A Fig) or the MOI (10–50) (S4B Fig). Taken together, these data indicated that *rv0180c* is critical for successful invasion of macrophages. This led us to examine the intracellular fate of the three strains.

To this end, hMDMs were infected with GFP-expressing WT, Δ*rv0180c*::*km* mutant, or Δ*rv0180c*::*km* complemented strains at an MOI of 2:1 for 2h at 37°C. They were then fixed at 2h and 144h post-infection (p.i.) and analyzed by confocal microscopy (Fig 4D, 4E, and 4F). As depicted on Fig 4E, the distribution of GFP fluorescence intensity per infected cell was not significantly different for the three strains at 2h p.i. although the difference in the percentage of infected cells was maintained in these conditions (Fig 4F). In contrast, this distribution was shifted to the lower values for the Δ*rv0180c*::*km* mutant in comparison to the two other strains at 144h p.i. (Fig 4E). These results established that *rv0180c* is required for intracellular multiplication of *M*. *tuberculosis* H37Rv in hMDMs.

*M*. *tuberculosis* is mostly taken up by macrophages through phagocytosis, a complex process involving the recognition and adhesion of bacteria through several phagocytic receptors and the subsequent remodeling of the actin cytoskeleton to promote bacterial engulfment. To explore the molecular mechanisms associated with the lower infectivity of Δ*rv0180c*::*km* mutant, we first examined the possible contribution of Rv0180c in the adhesion of bacteria to macrophages. We compared attachment of GFP-expressing WT, Δ*rv0180c*::*km* mutant or Δ*rv0180c*::*km* complemented strains to host cells by incubating bacteria with hMDMs at MOI 10:1 for 30 min at +4°C to allow bacteria to bind, whilst preventing uptake. We found around 4% cells having bound 1 bacteria per cell for the WT or the Δ*rv0180c*::*km* complemented strain but only 2% cells for the Δ*rv0180c*::*km* mutant (S5A Fig). These results suggested that disruption of the *rv0180c* gene affects the binding of *M*. *tuberculosis* to hMDMs. Then, we demonstrated that actin filament network was required for entry of WT and Δ*rv0180c*::*km* mutant into macrophages. Indeed, treatment of cells with cytochalasin, an actin-depolymerizing drug, similarly inhibited the internalization of both strains in a dose-dependent manner (S5B Fig). Difference between the strains was maintained at 0 or 0.1 μg/ml cytochalasin and inhibition of internalization was almost total for all strains at 1μg/ml cytochalasin.

**Fig 5:**
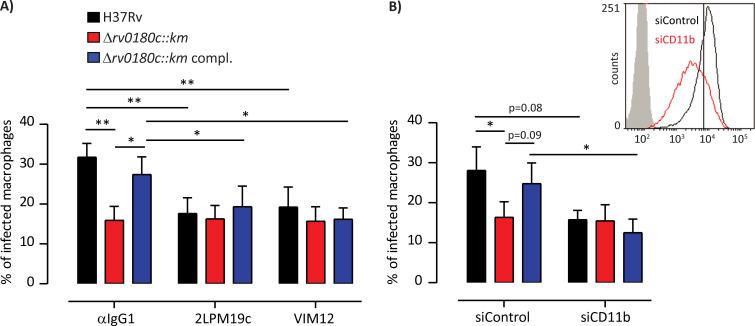
The *rv0180c* gene allows invasion of hMDMs via CR3. (A) hMDMs were pretreated for 30 min with 10 μg.ml^-1^ mAb directed against the I-domain (clone 2LPM19c) or the lectin-site (clone VIM12) of human CR3, or normal mouse IgG1 used as control or (B) hMDMs were transfected with non-targeting control siRNA or CD11b-targeting siRNA. Insert showed cytofluorimetric analysis of membrane expression of CD11b from a representative CD11b silencing experiment in hMDMs. (A,B) hMDMs were then incubated with either the parental H37Rv, the Δ*rv0180c*::*km* mutant, or the Δ*rv0180c*::*km* complemented strains at MOI 10:1 for 60 min at 37°C, fixed and prepared for analysis by confocal fluorescence microscopy. Vertical bar plots represented the percentage of infected cells. The values are means +/- SEM calculated from 4 (A) and 3 (B) donors. The significance of difference between strains was evaluated using the one-way ANOVA followed by Bonferroni’s post hoc test. The significance of difference between control and treatment was determined using the paired Student’s t-test; *p<0,05, ** p<0,01.

Finally we asked whether the lower infectivity of *Δrv0180c*::*km* mutant is due to a lower engagement of phagocytic receptors. Several membrane receptors have been described to participate in the invasion of macrophages by *M*. *tuberculosis* [37–39]. Here we focused on the complement receptor 3 (CR3) because this is the main receptor for entry of H37Rv in our model of hMDMs [36]. CR3 is a complex receptor with two binding domains, the I-domain and a carbohydrate binding lectin-like domain [40–43]. When we treated macrophages with the blocking anti-CD11b mAb directed against the I-domain of CR3 (2LPM19c), we observed a reduction of the percentage of infected cells for the WT and Δ*rv0180c*::*km* complemented strains, but not for the Δ*rv0180c*::*km* mutant and the difference for the three strains was annihilated (Fig 5A). This reduction was due to the specific blockade of CR3 since no effect was observed with the isotype control IgG1 (Fig 5A). These results were confirmed using CD11b siRNA knockdown. As shown in Fig 5B-insert, the transfection of hMDMs by siRNA targeting CD11b efficiently decreased the cell surface expression of CD11b when compared to the control siRNA. Under these conditions, we found that CD11b knockdown reduced the number of hMDMs infected with the WT and Δ*rv0180c*::*km* complemented strains without affecting cells infected with Δ*rv0180c*::*km* mutant (Fig 5B). Binding of *M*. *tuberculosis* to CR3 was found to involve the lectin site that recognizes capsular polysaccharides [44]. Therefore, we assessed the impact of treatment with an antibody, VIM12, raised against the lectin-like domain of CR3. Remarkably, pre-incubation with VIM12 reduced the percentage of cells infected with the WT and Δ*rv0180c*::*km* complemented strains but had no effect on cells infected with the Δ*rv0180c*::*km* mutant, as 2LPM19c (Fig 5A).

Altogether, these results showed that *rv0180c* is required for an optimal entry of *M*. *tuberculosis* in hMDMs through complement receptor CR3 and for intracellular replication. As binding of *M*. *tuberculosis* is decreased and as the CR3 lectin-like site is required, our results support the hypothesis that CR3-recognition of a bacterial carbohydrate located at the surface of H37Rv is impaired in the Δ*rv0180c*::*km* mutant.

### Rv0180c is required for resistance to non-specific stress and bacterial shape

**Fig 6:**
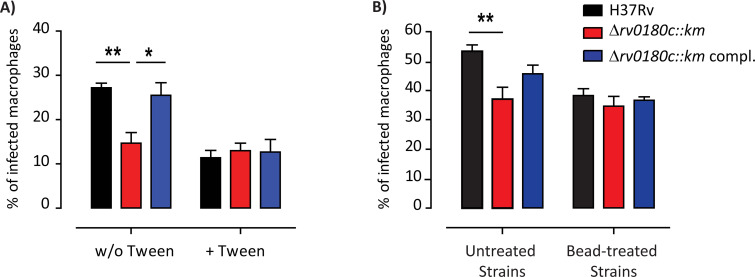
Removal of the bacterial capsule suppresses the differential uptake of the Δ*rv0180c*::*km* mutant. The parental H37Rv, the Δ*rv0180c*::*km* mutant, or the Δ*rv0180c*::*km* complemented strains were grown (A) in 7H9 ADC liquid medium with or without Tween-80 0.05% or (B) in Sauton liquid medium before removal of the capsule using agitation with glass beads. Untreated bacteria were used as control. (A,B) hMDMs were then incubated with bacterial preparations at MOI 10:1 for 60 min at 37°C, fixed and processed for analysis by confocal fluorescence microscopy. Vertical bar plots represented the percentage of infected cells. The values are means +/- SEM calculated from 3 donors. The significance of difference between strains was evaluated using the one-way ANOVA followed by Bonferroni’s post hoc test; *p<0,05, ** p<0,01.

If a CR3-ligand from the bacterial surface is missing in the Δ*rv0180c*::*km* mutant, removal of the capsular layer of the cell envelope should suppress the differential uptake. We used two different strategies to strip the bacterial surface. First, we cultured bacteria in the absence or the presence of detergent Tween-80 which was previously demonstrated to remove the capsule of mycobacteria [45]. We then compared uptake of the three strains by hMDMs (Fig 6A). We found that addition of detergent in the culture medium had no effect on the percentage of cell infected by the Δ*rv0180c*::*km* mutant. In sharp contrast, stripping the surface of the WT or Δ*rv0180c*::*km* complemented strains with Tween-80, decreased the infectivity of these strains and abolished the difference with the Δ*rv0180c*::*km* mutant. Second, we cultured the bacteria without detergent but we mechanically eroded the bacterial surface by agitation using glass beads. This treatment was previously found to preserve the bacterial viability whilst removing the capsule [46]. We then evaluated the impact of this mechanical treatment on the bacterial infection of hMDMs (Fig 6B). Our results showed that bead treatment abolished the difference between the Δ*rv0180c*::*km* mutant and the WT or Δ*rv0180c*::*km* complemented strains.

These findings suggest that difference in composition of the capsule may explain the lower infectivity of the Δ*rv0180c*::*km* mutant in comparison to the WT strain. We therefore comparatively analyzed the extract recovered after bead treatment. The outermost *M*. *tuberculosis* layer is mainly composed of polysaccharides (glucan, arabinomannan (AM) and mannan) and proteins, with small amounts of lipids [46–48]. We performed immunoblot experiments using either anti-glucan (IV56B6) or anti-AM/LAM antibodies, which recognize either the arabinan domain (F30.5 and CS-35) or the α(1->2)-linked mannose caps (55.92.1A1) [49,50]. We observed no significant differences between the Δ*rv0180c*::*km* mutant and the WT or Δ*rv0180c*::*km* complemented strains with any of the four antibodies (S6A Fig), suggesting that the overall amount and structure of polysaccharides is similar at the surface of the three strains. We also analyzed the lipids in the bacterial cell surface fractions obtained by bead treatment using High-Performance Thin Layer Chromatography (HP-TLC) with different solvent systems. Again we did not detect any difference between the Δ*rv0180c*::*km* mutant and the WT or Δ*rv0180c*::*km* complemented strains (S6C Fig). Finally, we performed proteomic analysis on the cell surface extracts. Only 5 proteins were differentially found in the extract from the Δ*rv0180c*::*km* mutant in comparison to that from the WT and *rv0180c*::*km* complemented strains (S7 Fig): Rv0108c, Rv0863, Rv1155A and Rv0180c were detected at significantly higher level in the WT and complemented strains than in the mutant and Rv3279c was found at significantly higher quantity in the mutant than in the WT or complemented strains. With the exception of Rv3279c, which encodes the protein BirA involved in biotin transfer onto acceptor proteins, none of the detected proteins exhibits a known function. None of these proteins were known CR3-ligand and none of the corresponding genes were detected in our TnSeq analysis suggesting that their differential expression is not associated with the lower infectivity. Taken together, these results suggested that none of the major molecules forming the capsule of *M*. *tuberculosis* is missing in the Δ*rv0180c*::*km* mutant in comparison to the parental or the Δ*rv0180c*::*km* complemented strains. Consistently, electron microscopy images reveal similar envelope layers in the WT strain and the Δ*rv0180c*::*km* mutant (S8 Fig).

**Fig 7:**
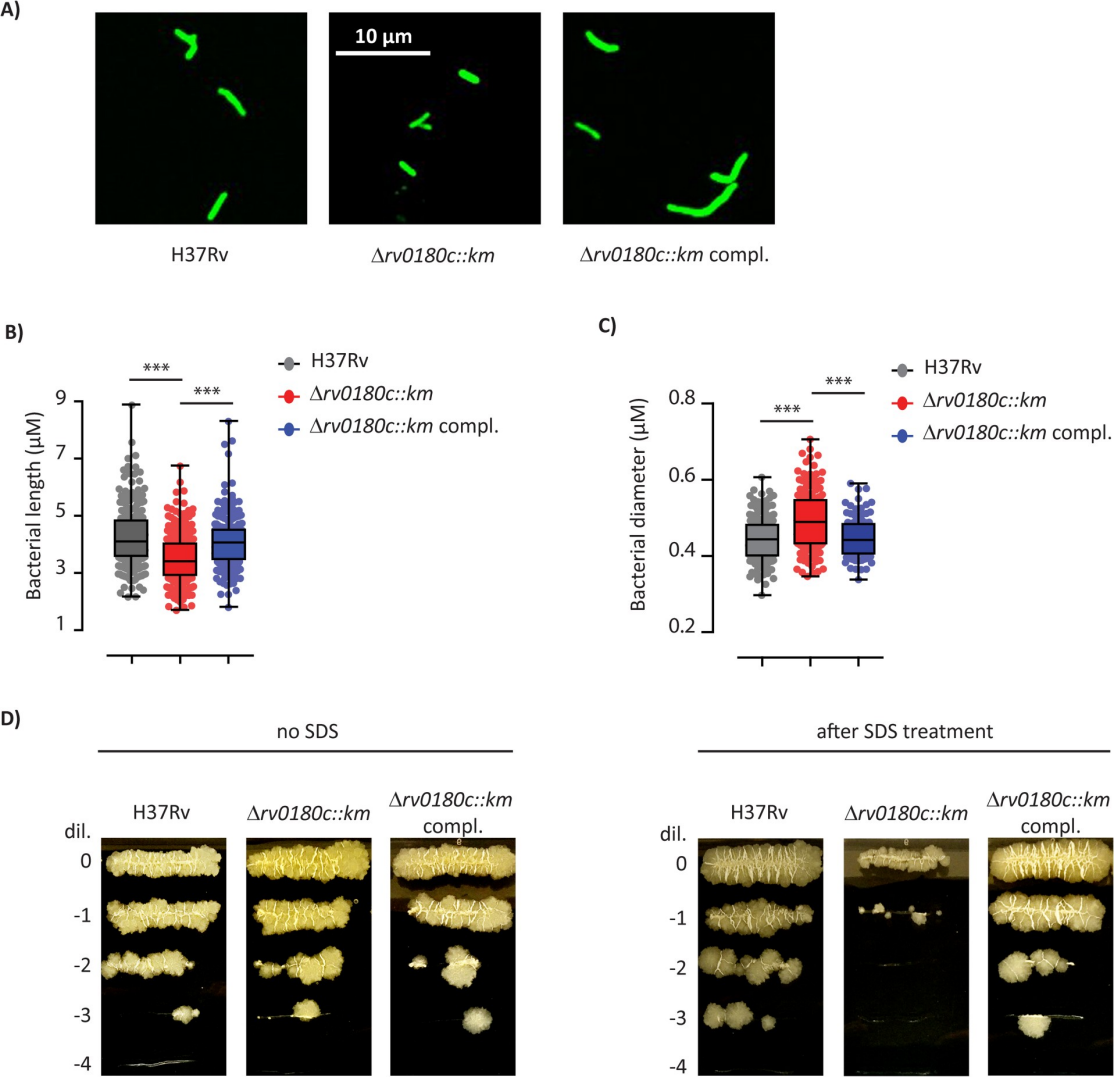
The Δ*rv0180c*::*km* mutation impacts the bacterial cell shape and cell envelope function. (A) Fluorescence microscopy image of H37Rv (WT), the Δ*rv0180c*::*km* mutant, and the Δ*rv0180c*::*km* complemented strains grown for 10 days in 7H9 ADC liquid medium. (B) Distribution of bacterial length for the parental H37Rv, the Δ*rv0180c*::*km* mutant and the Δ*rv0180c*::*km* complemented strains. Between 140 and 220 bacterial cells from two experiments were analyzed. (C) Distribution of bacterial diameter for the parental H37Rv, the Δ*rv0180c*::*km* mutant and the Δ*rv0180c*::*km* complemented strains. Around 80 bacterial cells from two independent experiments were analyzed. For (B) and (C), the statistical significance was evaluated using the Kruskal-Wallis rank sum test followed by Dunn’s multiple comparison test: *** p<0.001. (D) Impact of incubation with detergent on the viability of H37Rv, the Δ*rv0180c*::*km* mutant, and the Δ*rv0180c*::*km* complemented strains. Bacteria grown in 7H9 ADC to exponential phase (OD_600nm_ between 0.6 and 0.8) were incubated with or without 0.1% SDS during 4 days before spotting 5μl of serial dilution on 7H11 OADC agar plate.

Another explanation for the impaired CR3-mediated phagocytosis might be an altered morphology of bacteria that could affect the receptor activity. Indeed, physical properties of target can regulate phagocytosis (for review, see [51]). In addition, a mechano-sensitivity of CR3-dependent phagocytic uptake was recently reported [52]. This led us to examine further the morphology of WT and mutant strains. The fluorescent microscopy images suggested that the mutant strain is smaller than the WT or the complemented strains (Fig 7A). We therefore measured the length of bacteria (Fig 7B) and we found that the distribution of bacterial length was shifted toward the smaller size for the Δ*rv0180c*::*km* mutant in comparison to H37Rv and the complemented strains (Fig 7B). In contrast, electronic microscopy experiments indicated that the diameter was larger for the Δ*rv0180c*::*km* mutant than for the WT strain or complemented strain (Fig 7C). Taken together, these data indicated that the deletion of *rv0180c* affects the shape of *M*. *tuberculosis*. As the bacterial shape is determined by the cell envelope ultrastructure, we explored the functionality of this barrier by testing the sensitivity of the WT or mutant strains to stresses such as antibiotics or a detergent. First, we examined the minimum inhibitory concentrations (MIC) for rifampicin, a large polyketide that inhibits RNA polymerase, isoniazid and ethambutol, which block the biosynthesis of mycolic acids, and vancomycin that targets peptidoglycan synthesis. We found no difference in the MIC for the WT and the Δ*rv0180c*::*km* mutant (S1 Table). In contrast, when we incubated the strains for 1 or 4 days with 0.1% SDS, we found no difference after 1 day of treatment but ~10 fold less cfu for the Δ*rv0180c*::*km* than for the H37Rv parental strain after 4 days. Genetic complementation with the *rv0180c* gene fully restored resistance to SDS to WT level (Fig 7D).

Together, these results indicate that the overall composition of the bacterial outermost layer is conserved in the Δ*rv0180c*::*km* mutant but that inactivation of *rv0180c* impairs the cell envelope properties, with consequences on the sensitivity to SDS and the bacterial shape.

## Discussion

The functions and adaptations that increase the capacity of *M*. *tuberculosis* to seed in the lung of naïve person and to initiate an infection are major issue to understand what makes this bacterium such a successful human pathogen. In this study, we deployed a TnSeq global approach to identify mutants that exhibit defect in their capacity to initiate an infection in the lung of mice following intranasal infection. Consistent with the established key role of the *M*. *tuberculosis* cell envelope in pathogenicity [53,54], we found that 6 out of the 17 identified genes are required for the biosynthesis or translocation of lipids or lypoglycans from the cell envelope. Other known virulence factors, such as the regulators PhoPR and EspR or proteins required for resistance to acid stress and cholesterol degradation, were also picked in our analysis. In addition to these well-known virulence factors, we also found 3 new candidates, *rv0180c*, *rv1179c* and *rv1592c*, not previously described and of unknown functions, among which *rv0180c*.

The *rv0180c* gene encodes a 452 amino acids protein with 6 predicted transmembrane segments indicating that the protein is likely to be embedded into the *M*. *tuberculosis* plasma membrane. This protein is highly conserved in all the slow growing mycobacteria including *M*. *leprae*, suggesting that it contributes to the pathogenic lifestyle. Although absent from many fast growing mycobacteria, the Rv0180c protein is not restricted to slow growing mycobacteria because proteins with high sequence similarities are found in bacteria from related genera such as Nocardia or Rhodoccoccus. Several studies analyzing genome variation among clinical isolates identified mutations in *rv0180c*, including deletion or single nucleotide change introducing a stop codon [55,56]. The isolates bearing these mutations were virulent and transmitted indicating that a functional Rv0180c protein is not an absolute requirement for the pathogenicity of *M*. *tuberculosis*. Consistent with this observation, we found that the Δ*rv0180c*::*km* mutant retained the capacity to replicate in the lungs and spleen of infected mice and in hMDMs. The occurrence of a functional Rv0180c would rather increase the infectivity, providing a selective advantage over other isolates for long-term association with human. This would explain why the orthologue of Rv0180c seems functional in *M*. *leprae* in spite of the major pseudogenization of its genome [57].

We found that Rv0180c enhances infection of human macrophages and we consider several molecular mechanisms: i) Rv0180c is accessible at the bacterial cell surface and acts as a ligand for the macrophage phagocytic receptor CR3, ii) this protein is required for the production, modification, or translocation of a surface compound which is the ligand important for host cell invasion, iii) the cell envelope composition is not influenced by the occurrence of Rv0180c but the organization of the various molecules is altered changing the bacterial surface and accessibility of host-receptor ligands, or iv) the change in bacterial shape associated with the Δ*rv0180c*::*km* mutation affects the receptor-mediated phagocytosis. As mentioned earlier, Rv0180c is likely to be an integral inner membrane protein. The largest predicted loop is 129 amino acids long and appears too short to cross the various cell envelope layers whose the thickness was measured at ~50nM [45]. Therefore, it is unlikely that Rv0180c acts as a direct ligand of CR3. In our analyses, we found no difference between the wild type and the Δ*rv0180c*::*km* mutant regarding the production of the major components (polysaccharides, proteins and lipids) of the *M*. *tuberculosis* capsule. In addition, electron microscopy imaging did not reveal any difference in the organization of the cell envelope layers. Although we cannot exclude that we missed a subtle change in the structure of a defined compound from the bacterial cell surface, we found no evidence for a role of Rv0180c in the composition or organization of the surface layer.

Alternatively, our results showing a modification of the bacterial shape support the last scenario that the change in bacterial shape associated with the Δ*rv0180c*::*km* mutation impairs phagocytosis of bacilli. The physical characteristics of the target particle, such as shape, size and rigidity contribute to the efficiency of mechanosensitive phagocytosis but the underlying mechanisms are poorly explored (for review, see [51]). Recently, Jamouillé and colleagues reported that the rigidity of target particle increases the efficiency of CR3-mediated internalization [52]. Bacteria are very stiff particles, with young’s modulus within the megapascal range [58]. This stiffness is usually attributed to the bacterial cell wall, which has also other functions such as maintaining the cell shape and protecting the bacteria from various stresses. Here, we observed that the Δ*rv0180c*::*km* mutant, which was sensitive to SDS, has some defects in the function of its cell wall. It is thus tempting to speculate that Rv0180c is directly or indirectly involved in cell wall mechanical properties and may thus promote the activity of mechano-sensitive receptors for a successful internalization of *M*. *tuberculosis*.

So, this study shed light on a novel *M*. *tuberculosis* factor that modulates the infectivity in mice and human cells. The exact molecular mechanisms underlying the phenotype associated with the *rv0180c* deletion remains to be established but our results indicate that the Δ*rv0180c*::*km* mutant displays an altered cell envelope. As a main determinant of the bacterial shape is peptidoglycan, a next step would be to analyze it in the wild type and Δ*rv0180c*::*km* mutant. However, *rv0180c* is absent in many mycobacterial species and in some *M*. *tuberculosis* isolates indicating that, if Rv0180c contributes to modulate the peptidoglycan structure, it has to be subtle. In line with this proposal, recent data indicated that the length of mycobacterial galactan ^_^ a polysaccharide which covalently linked the peptidoglycan and arabinan ^_^ can affect cell morphology and resistance to environmental stresses [59]. The bacterial shape affects the progression of disease caused by many pathogens (for review, see [60]). The elucidation of genes that participate in the morphology of *M*. *tuberculosis* could provide a mean to explore new mechanisms involved in the pathogenesis of tuberculosis.

## Materials and methods

### Ethics statement

Animal studies were conducted following the CNRS guidelines for housing and care of laboratory animals. All protocols were reviewed and approved in compliance with the European Community council directive (EEC guidelines) and its implementation in France and received approval from the French Ministry for High Education and Research (number 201508271122464v2 (APAFIS#1535)).

Whole blood from donors was provided by Etablissement Français du Sang (EFS, Toulouse, France, under contract 21/PLER/TOU/IPBS01/2020-025). According to article L1243-57 of the French Public Health Code, the contract was declared to the French Ministry of Science and Technology (declaration number DC 2012–1715). Written informed consents were obtained from the donors before sample collection.

### Bacterial strains, plasmids, and growth conditions

The *M*. *tuberculosis* H37Rv Pasteur strain [61] was used for the transposon library and *Δrv0180c*::*km* mutant construction. Mycobacteria were grown at 37°C in Middlebrook 7H9 broth (Difco) supplemented with 10% (v/v) ADC without Tween-80 or in Sauton liquid medium. When required, kanamycin (40μg/ml), hygromycin (50μg/ml), streptomycin (20μg/ml), or Tween-80 0.05% final were added to the culture medium.

For the construction of the *Δrv0180c*::*km* mutant in *M*. *tuberculosis* H37Rv, two DNA fragments (1kb each) located upstream or downstream the *rv0180c* gene were amplified by PCR using primers 80a and 80b or 80c and 80d respectively. The two fragments were fused, generating an EcoRV restriction site at the fragment junction and amplified by PCR using primers 80e and 80f located inside the upstream and downstream region respectively. The PCR fragment was cloned into the plasmid pJET (ThermoFisher Scientific). A 0.8kb PCR fragment containing the kanamycin (*km*) gene was amplified from plasmid pET28a (Addgene) using primers km1 and km2 and inserted into the EcoRV site. The substrate for allelic exchange was produced by PCR amplification of a 2.8kb fragment using primers 80e and 80f. This DNA fragment was electroporated into a recombinant H37Rv strain expressing the recombineering system [62] from plasmid pJV53H and transformants were selected on km-containing plates. Twelve km-resistant clones were randomly picked and analysed by PCR using primers 80a+80g, 80d+80h, 80a+kmR, and 80d+kmF. One clone giving the expected PCR profile was selected for further experiments and named PMM292 (or *Δrv1080c*::*km*).

For complementation of the *Δrv1080c*::*km* mutant, the *rv0180c* gene was amplified from *M*. *tuberculosis* H37Rv using primers 80i and 80j and inserted into plasmid pJET. The *rv0180c* gene was then recovered on a NdeI and SpeI restriction fragment and inserted between the NdeI and SpeI sites of a derivative of the integrative plasmid pMV361 [63] carrying a hygromycin resistance gene and the mycobacterial promotor pBlaF* [64], to give the complementation plasmids pWM382. This plasmid was transformed into strain *Δrv1080c*::*km* to give PMM292::pWM382 (the *Δrv1080c*::*km* complemented strain). For macrophage infection, the H37Rv, *Δrv1080c*::*km* and the *Δrv1080c*::*km* complemented strains were transformed with plasmid pWM251, which is a replicative plasmid carrying a streptomycin resistance cassette and the GFP expressed from the mycobacterial promotor pBlaF*.

The primers used in this study are listed in S2 Table.

### Mouse infection

Female BALB/c mice, 8 weeks old, were purchased from Janvier (Le Genest St Isle, France) and housed in the IPBS ASB3 animal facilities.

For inoculum preparation, bacteria were grown at 37°C in 7H9 ADC with Tween-80 0.05% for 10 days. Before infection of mice, the bacterial clumps were removed by allowing the bacterial suspensions to sediment for 10 min and by centrifuging the supernatant for 8 min at 500xg. Bacteria count was determined using the 600nm optical density of the suspension. An optical density of 0.1 corresponds to 10^7^ bacteria per ml.

Mice were infected with 10^2^, 10^3^, 10^4^ or 10^6^ bacteria in PBS-Tween 0.05% via the intranasal or the intravenous route. At the indicated time points after infection, mice were euthanized and bacterial load was assessed in lungs and spleens. Organs were homogenized in 5 mL of PBS containing 0.05% Tween-80 using a GentleMACS dissociator and serial dilutions were plated onto solid 7H11 OADC medium. The numbers of CFU were evaluated 21 days later.

### Transposon library and *in vivo* screen

The transposon library used in this study were generated in the *M*. *tuberculosis* strain H37Rv using the phagemid ϕMycomarT7 [13]. Approximately 7x10^4^ clones were pooled to generate the H37Rv transposon library and aliquots of this library were stored at -80°C. Prior to infection, an aliquot of the library was inoculated into 50ml liquid medium and incubated at 37°C for 2 weeks. Part of this culture was diluted in PBS Tween-80 0.05% to reach a final concentration of 2.5x10^7^ cfu/ml and to generate the inoculum. The remaining part of the culture was used for genomic DNA extraction and TnSeq analysis. Eight BALB/c female mice, 7–8 weeks old, were infected intranasally with 40μl (10^6^ cfu) of the library. After 1 day or 8 days of infection, four mice (biological replicates) were euthanized. Lungs were collected and homogenized in 5ml PBS Tween-80 0.05% using a GentleMacs Dissociator apparatus (Miltenyi Biotec). A fraction of the lung homogenates (1ml) was used for bacterial load evaluation. Cfu in each sample was evaluated by plating serial dilution of lung homogenates on 7H11 OADC Km plates. The remaining fraction (4ml) of each lung homogenate was centrifuged at 90 g for 5 min to remove tissue debris. The supernatant was transferred to 2 ml tubes and centrifuged at 17000xg for 10 min to pellet bacteria. The bacterial pellet was resuspended in 5 ml 7H9 ADC 0.05% Tween-80 Km and incubated at 37°C for 20 days before genomic DNA extraction.

### Genomic DNA isolation, sequencing and TnSeq analysis

For each sample, the bacterial culture was split in two parts and two genomic DNA extractions were performed using the DNeasy kit (Qiagen). DNA was resuspended in 120μl Elution buffer. The DNA library preparation was performed at the GeT-PlaGe core facility (INRAE, Toulouse) using the TruSeq DNA Sample Prep Kit v2 (Illumina). Briefly, 200 ng of genomic DNA was fragmented to 550 bp pieces by sonication on M220 Focused-ultrasonicator (COVARIS). The DNA fragments were purified using SPB beads (kit beads) and the 3’ ends of the blunt fragments were mono adenylated. SP2-Bottom and SP2-top (S2 Table) were previously combined and diluted in AB 1X Buffer to be annealed (95°C, 2min; room temperature 45 min) to form adapter SP2. Adapter SP2 was ligated to fragments and transposon–junctions were enriched by PCR amplification for 1 cycle (94°C, 60s) followed by 25 cycles (94°C, 30s; 65°C, 60s; 72°C, 90s) and 1 cycle (72°C, 10min) using the primer P5-SP1-Tn recognizing the transposon end and a primer P7-index-SP2 recognizing the SP2 adapter and introducing the index for Illumina sequencing (S2 Table). For each DNA sample, five independent PCR amplification were performed and sequenced (technical replicates). Size selection was performed on the PCR product. Library quality was assessed using an Advanced Analytical Fragment Analyzer and libraries were quantified by qPCR using the Kapa Library Quantification Kit (Roche). DNA-sequencing was performed on an Illumina HiSeq3000 using a paired-end read length of 2x150 pb with the Illumina HiSeq3000 Reagent Kits. More than 5 millions reads were generated for each sample. The sequencing reads were aligned to the H37Rv reference genome [61], and unmapped, unpaired reads, and PCR duplicates were removed using BWA and Samtools softwares. The sequencing reads were analyzed to identify the Himar inverted terminal repeat and the adjacent TA using a custom script (available upon request) allowing one mismatch. The sequence following the TA insertion site was aligned to the H37Rv genome sequence to identify the TA dinucleotide insertion site coordinates. For each sequencing data set, a table was generated containing the number of read for each TA insertion site.

For TnSeq analysis, each insertion count from mouse samples was compared to the insertion count obtained with the inoculum sample using both the ESSENTIAL [18] and TRANSIT [19] methods. For each comparison and each method, a list of genes with significant different insertion count (adjusted p-values <0.05 and Log2 Fold change <-2 or >2) was generated. Genes identified in three out of four comparisons using the same statistical analysis method were retained. We then selected only the genes retained using the two methods to produce two short lists of genes with differential insertion count in comparison to the inoculum after 1 day or 8 days in mice (S1 Fig).

### Antibodies and reagents for hMDMs experiments

RPMI 1640 and glutamine were from Gibco. The 2LPM19c and the VIM12 mouse antibodies directed against the CD11b subunit of human CR3 or against the lectin-site (IgG1, dilution 10μgmL^-1^) and normal mouse IgG1 were purchased from Santa Cruz Biotechnology. DAKO mounting medium was from DAKOCytomation. The other reagents were purchased from Sigma-Aldrich.

### hMDMs culture, infection, staining and imaging

Peripheral blood mononuclear cells and human monocyte-derived macrophages (hMDMs) were prepared as previously described [65]. Briefly, hMDMs were generated from monocytes cultured for 7 days on glass coverslips in 24-well tissue culture plates (4.10^5^ cells/well) containing RPMI 1640 supplemented with 2mM glutamine and 7% heat inactivated human AB serum.

Single bacteria suspensions were prepared from exponentially growing strains as previously described [36]. Briefly, bacteria were grown for 10 days in 7H9 ADC without Tween. The bacterial suspension was centrifuged at 1000xg for 10 min at room temperature. The medium was discarded and the bacteria were dispersed by shaking for 1 min with glass beads (3mm diameter) and resuspended in serum-free RPMI 1640 medium. The remaining clumps were removed by allowing the bacterial suspensions to sediment for 10 min and by centrifuging the supernatant for 10 min at 200xg. Bacteria count was determined from the optical density at 600nm (OD_600nm_ = 1 for 2x10^7^ bacilli/ml).

Infection of macrophages and phagocytosis were assessed as previously described [36]. Briefly, cells were infected with GFP-expressing mycobacteria at the appropriate multiplicity of infection (MOI) in serum-free RPMI 1640 medium and infection was allowed to proceed for 30 min at 4°C for binding or for different times at 37°C for uptake (30, 60, or 180 min). When indicated, hMDMs were pretreated for 30 min with 10 μg ml^-1^ mouse antibodies (mAb) directed against the I-domain (clone 2LPM19c) or the lectin-site of human CR3 (clone VIM12), or normal mouse IgG1 used as control. At the end of infection, extracellular bacteria were removed by three successive washes with fresh medium. At the indicated time points, cells were fixed with 3.7% PFA. Coverslips were then quenched with 50mM NH_4_Cl in PBS for 2 min and permeabilized with 0.03% triton X100 for 10 min before washing 3 times with PBS. Nuclei and F-actin were labelled with DAPI (1/1000) and with rhodamine-phalloidin antibodies (1/2000) (Life Technologies), respectively. Coverslips were mounted on glass slides using mounting medium and samples were imaged using a confocal Spinning Disk microscope (Olympus, IX83) equipped with a Yokogawa X1 head and an EMCCD camera (iXon Ultra, Andor). The system was driven by Andor IQ_3_ software. Images were taken using a 60X oil immersion objective (1.35 NA, UPlanSAPO) and acquisition was performed using the following settings: scanning mode xyz, sequential color acquisition and pixel resolution 1,024 x 1,024.

We then evaluated bacterial infectivity. To this end, images were analyzed using macros (available upon request) and FIJI (https://imagej.net/Fiji), as previously described [66]. Briefly, images were split into their constitutive color channels and a z-projection summing the slices was used to visualize bacteria either bound at the surface of macrophages or ingested. We then enumerated the number of intracellular bacteria per cells and the percentage of macrophages having bound or ingested bacteria. The location of the individual cells was done by calculating the center of mass from the DAPI nuclei image. The region of interest (ROI) of individual cells was determined from the nuclei center of mass using the Voronoi network analysis. ROI was applied to the green channel image, corresponding to the GFP signal from the bacteria, and GFP fluorescence intensity (GFP FI) corresponding to the sum of all GFP-positive pixels (RAWIntDen), was determined and quantified per infected cell. Values obtained for each lot of hMDMs were normalized by subtracting the mean fluorescence intensity value observed with H37Rv control for each experiments and by adding the mean fluorescence intensity value observed for all experiments with H37Rv.

Each experiment was performed at least two times independently with cells from 3 independent donors or more and more than 100 cells from different fields were analyzed.

### hMDM siRNA transfection

hMDMs were transfected with 200 nM non-targeting control siRNA or pre-designed ON-TARGETplus SMARTpool siRNA targeting human CD11b (GE-Dharmacon) using HiPerFect transfection reagent (Qiagen), as described previously [67,68]. Briefly, siRNAs were mixed with HiPerFect transfection reagent and serum-free RPMI medium and incubated at room temperature for 15 min. The lipid-siRNA complexes (125 μl) were added drop-wise onto the medium (250 μl) of day 5 hMDMs and incubated for 6 h at 37°C and 5% CO_2_. After transfection, 500 μl of RPMI containing 7% autologous serum was added and cells were incubated at 37°C and 5% CO2 for additional 72 h before infection. Efficiency of gene knockdown was assessed by cytometry using RPE-conjugated anti-human CD11b mAb (clone 2LPM19c).

### Bacterial resistance to SDS and antibiotics

For SDS sensitivity assays, bacterial cultures were inoculated in 7H9 ADC at dilution 1:100 using 3 weeks old precultures and incubated for 10 days to reach an OD_600nm_ between 0.6 and 0.8. The bacteria were then diluted to an OD_600nm_ of 0.1 in 7H9 ADC supplemented with 0.1% SDS and incubated for 1 and 4 days at 37°C. Serial dilutions of these cultures were spotted on 7H11 OADC.

For evaluation of MIC, bacterial cultures were inoculated in 7H9 ADC Tween 0.05% at dilution 1:100 using 3 weeks old precultures and incubated for 10 days. The bacteria were then diluted to an OD_600nm_ of 0.01 in 5ml 7H9 ADC Tween 0.05% in McFarland tubes, and incubated in the presence of various concentrations of antibiotics: isoniazid (INH) and Ethambutol (ETH): 5, 2.5, 1.25, 0.625, 0.312, 0.156, 0.078, 0.039, 0.02, 0 μg/ml; Rifampicin (RIF): 0.2, 0.1, 0.05, 0.025, 0.012, 0.006, 0.003, 0.0015, 0.0008, 0 μg/ml; Vancomycin (VANCO): 100, 50, 25, 12.5, 6.25, 3.125, 1.562, 0.781, 0.391, 0 μg/ml. At 9 or 11 days of incubation, each tube was vortexed before evaluation of turbidity using a McFarland densitometer. The experiment was performed in triplicate. The MIC indicated in S1 Table corresponds to the lowest antibiotic concentration for which no growth was observed.

### Analysis of bacterial size and diameter and electron microscopy

To measure bacterial length, mycobacteria were grown at 37°C in 50mL 7H9 ADC broth without Tween-80. After ten days of culture, the bacterial suspension was centrifuged at 1000xg for 10 min at RT before medium was discarded. Bacteria were dispersed by shaking for 1 min with glass beads (3mm diameter) and fixed 2 hours with PFA 3.7%. After centrifugation, PFA was discarded and bacteria were resuspended in PBS and mounted between glass slides and coverslips using mounting medium. Bacterial length was determined using FIJI software.

To determine bacteria diameter, mycobacteria were directly fixed with fixation buffer (PFA 4%, glutaraldehyde 5% (v/v)) 15 min at RT before centrifugation at 4000xg 8 min. The pellet was resuspended in fixation buffer (PFA 2%, glutaraldehyde 2.5%) 2 hours at RT temperature and post-fixed with 1% OsO4 in the same buffer. Samples were concentrated in agarose and treated for 1 h with 1% aqueous uranyl acetate. Then, they were dehydrated in a graded ethanol series and embedded in EMBed-812 resin (EMS). After 48 h of polymerization at 60°C, ultrathin sections (80 nm thick) were mounted on 200 mesh formvar-carbon coated copper grids. Sections were stained with Uranyless (Delta Microscopies) and 3% Reynolds lead citrate (Chromalys). Grids were examined with a TEM (Jeol JEM-1400, JEOL Inc) at 80 kV. Images were acquired using a digital camera (Gatan Orius, Gatan Inc, Pleasanton, CA, USA) at a 25K magnification.

For the Cryoelectron microscopy (Cryo-EM), the bacteria were fixed with fixation buffer (PFA 4%, glutaraldehyde 0.2% (v/v)) 30 min at RT before centrifugation at 4000xg 8 min. The pellet was then discarded and bacteria resuspended with PBS. For freezing, 3 μL of sample were deposited onto glow-discharged lacey carbon grids and placed in the thermostatic chamber of a Leica EM-GP automatic plunge freezer, set at 20°C and 95% humidity. Excess solution was removed by blotting with Whatman n°1 filter paper for 0.5 seconds, and the grids were immediately flash frozen in liquid ethane at -185°C. The frozen specimens were placed in a Gatan 626 cryo-holder, and Cryo-EM was carried out on a Jeol 2100 microscope, equipped with a LaB_6_ cathode and operating at 200 kV, under low dose conditions. Images were acquired with SerialEM software, with defocus of 1–2 μm, on a Gatan US4000 CCD camera. This device was placed at the end of a GIF Quantum energy filter (Gatan, Inc.), operated in zero-energy-loss mode, with a slit width of 25 eV. Images were recorded at a nominal magnification of 4000 corresponding to calibrated pixel sizes of 1.71Å.

### Production of bacterial bead-fraction

To extract the capsular layer of the bacterial cell envelope, we grew a 110ml culture in liquid Sauton medium for each strain (H37Rv:pWM251, PMM292 (*Δrv1080c*::*km)*:pWM251, and PMM292::pWM282:pWM251). After 15 days, 10ml were taken from each culture for phagocytosis experiment in hMDMs. The remaining 100ml were centrifuged at 2500xg for 10min. Equivalent volumes of glass bead (3mm) were added to the bacterial pellets in addition to 1.2 ml of PBS. The samples were vortexed for 4min. After 5 min sedimentation, 1.1ml of supernatant was collected and split into two parts (100μl and 1000μl). The 100μl part was centrifuged for 8 min at 500xg to remove clumps and the supernatant was used for hMDMs infection. Bacteria count was determined using the OD_600nm_ of the suspension. The 1ml fraction was pelleted for 1min at 13400xg to remove the bacteria. The supernatant was collected and filtered twice on 0.22μm pore filters. This bead fraction was used for proteomic and dot blot analysis. The experiment was performed in triplicate.

### Dot-blot analyses of lypoglycans

Serial dilutions of the bead-fractions for WT, *Δrv1080c*::*km* mutant and *Δrv1080c*::*km-*complemented strains were spotted (2μl) on BioTrace nitrocellulose membrane (PALL Laboratory) and dried for 1h at 60°C before incubation overnight with PBS supplemented with Tween-20 0.1% and skim milk 10% at 4°C. The membrane was then incubated for 1h at room temperature with primary antibodies (anti-glucan (IV56B6), anti-AM (F30.5 and CS-35), and anti-ManLAM (55.92.1A1)) [49,50] diluted 1/1000 in PBS Tween-20 0.1% and skim-milk 1%. The membrane was washed 3 times in PBS Tween-20 0.1% for 15min before incubation with the secondary antibody, HRP Goat anti-Mouse (Biorad) diluted 1/2500 in PBS Tween-20 0.1% and skim milk 10% at 4°C overnight. After 3 washes, signals were revealed using HRP substrate (HRP Immobilon Western, Merck) for chemiluminescence and ChemiDoc Imaging system (Biorad).

### Proteomic analyses

100 μg of proteins from the bead extracts were dried in a SpeedVac (Thermo Fisher Scientific) and resuspended in 5% SDS. The protein solutions were reduced and alkyled with a 100mM TCEP, 400mM Chloro Acetamide solution for 5 min at 95°C. The proteins were acidified with a final concentration of 1.2% phosphoric acid and diluted with 7 volumes of S-Trap buffer (90% Methanol, 100 mM triethylammonium bicarbonate (TEAB), pH 7.1).The samples were then transferred into S-Trap Mini Spin columns (Protifi), trapped in filter by centrifugation at 4000xg for 1 min, washed 6 times with 400 μL of S-Trap buffer, and centrifuged at 4000×g for 1 min. Proteolysis was performed with the addition into the S-Trap cartridge of 1μg trypsin (125μl in 50 mM ammonium bicarbonate) and 1h incubation at 47°C. Peptide extraction was performed with 3 steps of elution: 80 μL ammonium bicarbonate 50 mM, 80μl 0.2% formic acid, and 80 μL of acetonitrile 50% /formic acid 0,2%, with centrifugations at 4000×g for 1 min. The peptide extracts were dried in a SpeedVac and resuspended with 22 μL of 5% acetonitrile and 0.05% trifluoroacetic acid. The peptides were analysed by nano-liquid chromatography–tandem mass spectrometry (MS/MS) using an UltiMate 3000 RSLCnano system (Dionex, Amsterdam, The Netherlands) coupled to an OrbiTrap Fusion mass spectrometer (Thermo Fisher Scientific, Bremen, Germany). Five microliters of each sample was loaded onto a C18 pre-column (300 μm id, 5 mm; Dionex), at 20 μl/min, in 5% acetonitrile and 0.05% trifluoroacetic acid. After 5 min of desalting, the pre-column was switched on line with the analytical C18 column (75 μm id × 50 cm C18 column; packed in-house with ReproSil-Pur C18-AQ 3 μm resin, (Proxeon Biosystems, Odense, Denmark), equilibrated in 95% solvent A (5% acetonitrile and 0.2% formic acid) and 5% solvent B (80% acetonitrile and 0.2% formic acid). Peptides were eluted using a 5–50% gradient of solvent B over 105 min and at a flow rate of 300 nL/min.

The OrbiTrap Fusion was operated in Data Dependent Acquisition mode to automatically switch between full scan MS and MS/MS acquisition using Xcalibur software (Thermo Fisher Scientific). Survey scan MS was acquired in the Orbitrap over the *m*/*z* 400–1500 range, with the resolution set to a value of 120,000 (m/z 400). The Top Speed mode (3s) was used to perform the DDA Aquisition, the most intense ions per survey scan were selected for higher-energy collisional dissociation, and the resulting fragments were analysed in the linear ion trap. Dynamic exclusion was employed within 60s to prevent repetitive selection of the same peptide.

Mass spectrometry raw files were analyzed using the Proline software version 1.6 [69]. MS/MS spectra were searched in the Mascot search engine (Matrix Science Inc., Boston, MA, USA) against the forward and reverse MTB H37Rv native 20201001 database. The digestion enzyme was set to trypsin/P with up to two missed cleavages. Methionine were searched as variable modifications and carbamidomethyl of cysteine as fixed modification. Parent peptide masses and fragment masses were searched with maximal mass deviation of 10 ppm and 20 ppm respectively. A first level of false discovery rate (FDR) filtration was done on the peptide-spectrum match level, and this was followed by a second level of FDR control on the protein level. Both filtrations were performed at a 1% FDR. These filtrations were done using a standard target-decoy database approach. For label-free relative quantification of the samples, the “match between runs” option of Proline was enabled to allow cross-assignment of MS features. The mass spectrometry proteomics data have been deposited to the ProteomeXchange Consortium via the PRIDE partner repository with the dataset identifier PXD024319.

### Lipid analyses

For lipid analysis of bead fractions, bacteria were grown in 200ml 7H9 liquid medium supplemented with 0.2% dextrose for 15 days. Each bacterial culture were splitted in 4 x50ml tubes and centrifuged at 2500xg for 10min. The supernatants were discarded and 12 glass beads (3mm) were added to each bacterial pellet. The samples were vortexed for 4min before addition of 2.5ml PBS. The 4 samples for each strain were pooled, transferred to a 15ml tube and centrifuged for 10min at 2500xg. The supernatants were collected (8ml) and incubated with 10ml CHCl_3_ and 20ml CH_3_OH. After 48h, 10ml CHCl_3_ and 10ml H_2_O were added and the organic phases were collected after 12h and dried. The lipid extracts were resuspended in 150μl CHCl_3_ and analyzed by HPTLC. The experiments were performed twice independently.

### Statistical analysis

All results are expressed as mean ± standard error of the mean (SEM), unless otherwise indicated. Information on the statistical tests used, and the exact values of n (number of donors or bacteria) are mentioned in the Figure legends. Statistical analyses were carried out using GraphPad Prism 5 or GraphPad Prism 9. The significant difference between individual groups was determined using two-tailed paired Student’s t test. For more than two groups, the variance among the groups was calculated using a non-parametric (Kruskal Wallis) or parametric one-way ANOVA test followed by a post-hoc test. P values lower than 0.05 were considered statistically significant.

## Supporting information

S1 Fig Identification of *M. tuberculosis* mutants with altered capacity to seed and initiate an infection in the lung of mice. (A) We generated a *M*. *tuberculosis* transposon mutant library and analyzed the number of TA site containing at least one transposon insertion in the library used to infect mice (corresponding to the inoculum) or after 1 day or 8 days of infection. The values in the table indicate that there is no substantial reduction in the number of different mutants in the library before and after infection. (B) The distribution of insertions in the bacterial populations recovered from each mouse at day 1 or day 8 was analyzed using TnSeq and compared with the distribution in the inoculum using the TRANSIT or ESSENTIAL methods. The figure shows Venn-diagrams of results obtained for each mouse and the two analytical methods. For each day, we retained hits identified in at least three mouse and the two methods. Finally, we retained only the 17 hits identified both at day 1 and day 8 post-infection.
(EPS)

S2 Fig Organization of the *rv0180c* locus in various mycobacterial species and construction of the Δ*rv0180c::km* mutant. (A) Schematic view of the *rv0180c* genomic locus in *M*. *tuberculosis* and the orthologous region in *M*. *marinum*, *M*. *leprae* and *M*. *smegmatis*. The percentage of amino-acid identity of encoded protein with the *M*. *tuberculosis* orthologue is indicated below each gene name. Grey genes in *M*. *leprae* correspond to pseudogenes. (B) Construction of the *M*. *tuberculosis* Δ*rv0180c*::*km* mutant. The primers used for characterization of the Δ*rv0180c*::*km* mutant are indicated. (C) PCR analysis of the Δ*rv0180c*::*km* mutant. (D) Growth of the parental *M*. *tuberculosis* H37Rv, the Δ*rv0180c*::*km* mutant and Δ*rv0180c*::*km* complemented strain in 7H9 ADC Tween-80 0.05% liquid medium. The OD_600nm_ was followed over a 16 days period.
(EPS)

S3 Fig The Δ*rv0180c::km* mutant is impaired for implantation in the lungs of mice after intravenous infection. Parental *M*. *tuberculosis* H37Rv and the Δ*rv0180c*::*km* mutant were used to infect mice intravenously using ~10^4^cfu per mouse. At 1, 8 and 21 days after infection, 4 mice for each group were sacrificed and the bacterial load in the lungs (A) and spleens (B) was evaluated by plating serial dilution of organ homogenate. Results are shown as the Log_10_ cfu for individual mice and the mean. **p<0.01 by one-way ANOVA followed by the Bonferroni post hoc test.
(EPS)

S4 Fig The Δ*rv0180c::km* mutant is impaired in its ability to infect hMDMs. hMDMs were incubated with GFP-expressing strains (parental H37Rv (WT), the Δ*rv0180c*::*km* mutant, or the Δ*rv0180c*::*km* complemented strain) and processed for counting the number of infected cells by fluorescence microscopy. (A) Vertical bar plots represent the percentage of hMDMs having ingested at least one bacterium after 30, 60 or 180 min of incubation at MOI 10:1. The values are means +/-SEM from 4 to 8 donors. The statistical significance was evaluated using the one-way ANOVA followed by the Bonferroni post hoc test. *p<0.05, **p<0.01. (B) Vertical bar plots represent the percentage of hMDMs having ingested at least one bacterium after incubation with bacteria at MOI 10:1 or 50:1 for 1h at 37°C. The values are means +/-SEM from 2 to 5 donors.
(EPS)

S5 Fig The Δ*rv0180c::km* mutant is impaired in its ability to bind and invade hMDMs through an actin-dependent process. (A) hMDMs were incubated with either the WT, the Δ*rv0180c*::*km* mutant, or the Δ*rv0180c*::*km* complemented strains at MOI 10:1 for 30 min at +4°C, fixed and processed for analysis by confocal fluorescence microscopy. (B) hMDMs were pretreated for 30 min with various concentration of cytochalasin and then put in contact with either H37Rv WT or the Δ*rv0180c*::*km* mutant at MOI 10:1 for 1h. Vertical bar plots (A) and dot plot (B) represent the percentage of hMDMs having ingested at least one bacterium. The values are means +/- SEM calculated from two donors. The statistical significance was evaluated using the one-way ANOVA followed by the Bonferroni post hoc test.
(EPS)

S6 Fig The lipid and glycoconjugate compositions of the outermost layer of the cell envelope of both the *M. tuberculosis* WT, the Δ*rv0180c::km* mutant, or the Δ*rv0180c::km* complemented strains are similar. (A) Dot-blot analysis of bead-fraction using various antibodies raised against glycoconjugates from the cell envelope: anti-glucan (IV56B6), anti-AM/LAM (F30.5 and CS-35, which recognize the arabinan domain and 55.92.1A1, which recognizes the α(1->2)-linked mannose caps) [49,50]. Capsular extracts of liquid cultures (recovered using agitation with glass beads) were spotted in 3-fold serial dilution steps onto nitrocellulose membrane before incubation with antibodies. Data shown are representative for 3 independent experiments. (B) Control experiment showing that the Dot-blot method detected a glucan deficient H37Rv-mutant. We used the *M*. *tuberculosis* H37Rv *ΔtreSΔglgC* double mutant deficient in capsular glucan synthesis [70]. Dot-blot analysis using anti-LAM or anti-glucan antibodies demonstrated lack of glucan production but synthesis of LAM or AM in the H37Rv *ΔtreSΔglgC* double mutant. (C) HP-TLC analysis of the lipid content of the fraction recovered after glass-bead treatment of *M*. *tuberculosis* WT, the Δ*rv0180c*::*km* mutant, or the Δ*rv0180c*::*km* complemented strains. The TLC were run in various solvent systems and lipids were visualized using copper sulfate in 8% phosphoric acid. The area were indicated *M*. *tuberculosis* lipids are migrating is marked. This experiment was performed twice independently.
(EPS)

S7 Fig The protein composition of the outermost layer of the cell envelope of both the *M. tuberculosis* WT, the Δ*rv0180c::km* mutant, or the Δ*rv0180c::km* complemented strain are similar. Volcano plot of log significance (-Log_10_ of T-Test p-value) versus Log_2_ of protein abundance ratio in the bead-fraction from the *M*. *tuberculosis* WT vs the Δ*rv0180c*::*km* mutant (A), the Δ*rv0180c*::*km* complemented strain vs the Δ*rv0180c*::*km* mutant (B), or the *M*. *tuberculosis* WT vs the Δ*rv0180c*::*km* complemented strain (C). The experiments were performed with three biological replicates for each bacterial strain. Peptides assigned to 2378 H37Rv proteins were identified. The threshold was fixed at 2 for the fold change and 0.05 for the p-value. Five proteins were detected at significant different level in the Δ*rv0180c*::*km* mutant in comparison to the WT and *rv0180c*::*km* complemented strains (Rv0180c, Rv0108c, Rv0863, Rv1155A, Rv3279c) and not in the comparison of the WT vs the *rv0180c*::*km* complemented strain.
(EPS)

S8 Fig Cell envelope of *M. tuberculosis* H37Rv and the Δ*rv0180c::km* mutant by Cryo-EM. Bacteria growth in 7H9 ADC were fixed, frozen and imaged by Cryo-EM. The plasma membrane (PM) and outer membrane (OM) are indicated. Representative images are presented and did not reveal alteration of the cell envelope layers.
(EPS)

S1 Table Minimal Inhibitory Concentration (MIC) of isoniazid, Ethambutol, Rifampicin and Vancomycin for the parental *M. tuberculosis* H37Rv (WT) or the *Δrv0180c::km* mutant. (DOCX)

S2 Table Name and sequence of oligonucleotides used in this study. (DOCX)

S1 Data Read counts for each TA position in the genome in the inoculum and each of the replicates, related to Figs 1 and S1. (XLSX)
