# Supplementary material for: Rv0180c contributes to Mycobacterium tuberculosis cell shape and to infectivity in mice and macrophages
Source: PLoS Pathog. 2021 Nov 1;17(11):e1010020. doi: 10.1371/journal.ppat.1010020 (PMC8584747; doi:10.1371/journal.ppat.1010020)
Supplement: S2 Table — (DOCX) [file ppat.1010020.s010.docx]

| **Name** | **Sequence (5’->3’)** | **Modification** |
| --- | --- | --- |
| SP2-bottom | GATCGGAAGAGC | 5’phosphorylation |
| SP2-top | GTGACTGGAGTTCAGACGTGTGCTCTTCCGATcT | Phosphorothioate |
| P5-SP1-Tn | AATGATACGGCGACCACCGAGATCTACACTCTTTCCCTACACGACGCTCTTC  CGATCTCCGGGACTTATCAGCCAACC |  |
| P7 index-SP2 | CAAGCAGAAGACGGCATACGAGAT-INDEX-GTGACTGGAGTTCAGACGTGT |  |
| 80a | ATCGGTTGGTCGAGCTGTTCA |  |
| 80b | ACGGTTCGGGATATCGGCTCCTGTCTGTCGTGG |  |
| 80c | ACAGGAGCCGATATCCCGAACCGTCACGCAGC |  |
| 80d | ATCGGTGTCACTACCCAGGC |  |
| 80e | ATGCCATCGTCACCCTCATTCAC |  |
| 80f | ATCGGAACAGGTCTTGGATGTT |  |
| 80g | CCGAATCGCTTTGACGTTCC |  |
| 80h | GCGAAAGCTCAAGCGAACAA |  |
| 80i | ATCATATGTCTCAAGCGCAGCCG |  |
| 80j | ATAACGTTTTACGGTTGTTCGCTTGAGC |  |
| km1 | GTCTGACGCTCAGTGGAAC |  |
| km2 | TTCAGGTGGCACTTTTCGG |  |
| kmF | GCCATCCTATGGAACTGCC |  |
| kmR | GCCTAGAGCAAGACGTTTCC |  |
